# A Leaflet Competition

**Published:** 1921-11

**Authors:** 


### CORRESPONDENCE.

[Correspondence on all subjects is invited, but we cannot in any way be responsible for the opinions expressed by our correspondents, who must give their name and address as a guarantee of good faith, but not necessarily for publication. Correspondents are reminded that brevity of style and conciseness of statement greatly facilitate early insertion.]

### "A Leaflet Competition."

*To the Editor of* THE HOSPITAL AND HEALTH REVIEW.

SIR,—It is in the tradition of your excellent journal to note defects of grammar, especially when they concern medical literature. The late and respected Dr. Charles Mercier used to delight your readers with correspondence upon the use of a passive verb with an object (e.g., "He was given a pill "), and the misuse of the verb to "develop" when applied to the symptoms of disease. Dr. C. Thackray Parsons entered the lists in this controversy, and, with characteristic humour, convicted Dr. Mercier, in one of his onslaughts, of having used the superlative when comparing two things.

Perhaps, then, I am in order if I remark upon the last sentence of the second paragraph of your announcement of "A Leaflet Competition." The offending sentence runs: "The Americans have told us that 'we dig our graves with our teeth,' and above all other 'minor ailments' at the present time the care of the teeth is the most important."

You somewhat weaken the admirable justice of the final remark by including "the care of the teeth" among "minor ailments"; of course accidentally. The idea of this competition is so excellent that any comment can serve only to emphasise its usefulness. What a writer of such leaflets Charles Dickens would have made!

Yours, SECRETARY.

